# Effect of COVID-19 on demand for healthcare in Togo

**DOI:** 10.1186/s13561-021-00335-x

**Published:** 2021-09-18

**Authors:** Yaovi Tossou

**Affiliations:** Member of the Research Centre for Economic Analysis of Public Policies (ANEPP), FASEG/University of Lomé, 01BP1515, Lomé, Togo

**Keywords:** COVID-19, Pandemics, Health care, Multinomial logit model

## Abstract

**Background:**

Demand-side barriers to health care are as important as supply-side factors in deterring patients from obtaining effective treatment during COVID-19. Developing countries, including Togo, have focused on reducing the risk of health care utilization during this period by ensuring basic health care services as an important policy to improve health outcomes and meet international obligations to make health services accessible.

**Methods:**

The data used to cover all 44 districts in the six (6) health regions of Togo, are from a national home survey. They are collected from July 8th to 17th, 2020. In each district chief town, a minimum of thirty (30) households were included by a systematic two-stage random draw (neighborhood and then home). Based on these data, the multinomial regression model was used to identify risk factors for the request for health care services during COVID-19.

**Results:**

A total of 1946 (with a response rate of 98.3%) participants were addressed in the study. The finding on households with age above 60 years indicated that the relative risk ratio (RRR = 23.97; 95% CI = 0.93; 615.38) allowed them to practice self-medication in lieu of modern healthcare facilities. The multinomial model revealed that the relative risk ratio of pre-COVID-19 activities (RRR = 4.87; 95% CI = 1.018; 23.38) permits households to rely on their self-medication choice and (RRR = 3.14; 95% CI = 0.91; 0.83) prefer public health facilities. Given that the head of the households (RRR = 0.19; 95% CI = 0.017, 2.11) is educated, he prefers the choice of private health centers during COVID-19 pandemic to public health facilities.

**Conclusion:**

This study has demonstrated that the majority (30.49%) of patients sought health care. The analysis shows that the loss of employment, activities before COVID-19 in households and areas not infected by the pandemics allow them to ask for health care (self-medication and public hospitals) despite the COVID-19 impacts. However, higher education and age determine a different choice of health care delivery by households. Thus, policy makers need to cast special emphasis on social policies to address home health shocks.

## Background

The COVID-19 occurs in the context of a global economic crisis, which highlights the health challenges and socio-economic factors, facing the most vulnerable people, in our communities. The demand for health care is characterized by the level of actual consumption of an individual in case of illness. This consumption of care differs according to factors of health care demand such as income, cost of care, education, social norms and traditions, and the quality and adequacy of services provided [1, 2]. It is from a multidimensional perspective that an individual makes a decision to reach a health care facility when he/she is suffering from a disease. Developing countries have focused on promoting the use of health care as an important policy to improve and meet international obligations to make health services more accessible. However, many policy initiatives depend on research, which focus on improving physical access rather than the utilization model of care services.

Thus, facing multiple measures pertaining to COVID-19, the demand for health care is likely to be reduced. This could have negative consequences for the health status of the population. These reductions can result from a range of supply and demand side factors. With this pandemic, most health care staff are being reassigned to respond to the COVID-19 emergencies, leaving the health care provider unable to meet the population’s request for care. Similarly, health facilities may be closed or have limited hours as well as supply chain disruptions may limit the availability of needed health products. Like demand-side factors, the implementation of social distancing policies and stay-at-home orders limit the movement of the population. This prevents households from choosing health facilities. Besides, the fears for COVID-19 and financial constraints reduce their ability to pay for care. Thus, [2] indicate that the consequences of social distancing measures and employment restrictions have had a much greater impact on several sectors, including the demand for health care. According to [3, 4], the COVID-19 pandemic has impacted income flows in the economy through reduced hours of work or closure. This resulted in a change in household income that was not conducive to health care demand during this pandemic.

However, the socio-economic status of households is one of the determining factors in the choice of health care services in case of illness. It is affected by both demand and supply side factors of health care. In terms of demand, many results show differences in the demand for care by households [1]. These socioeconomic factors are often assessed by income, occupation, education level, health insurance coverage, age, place of residence, etc. Some results show that lower socioeconomic backgrounds are more likely to not use health care by households during the COVID-19 pandemic. Several studies have shown that economic variables such as household income and price influence health care decision making, [5, 6] reveal that price, income, and distance from health centers to home are important determinants in households’ choice to use health care services. In addition, [7–10] have emphasized the importance of quality of care in promoting choice of health care services. Specifically, [11–13] have classified factors that influence demand for health care services into three categories such as predisposing factors (social characteristics), enabling factors (access to health care), and health care needs (characteristics of the perceived illness).

Furthermore, in the context of COVID-19, [14], find that the effect of physical removal measures, especially among people with chronic illnesses move them away from using health care services [14]. expressed concerns about access to health care. They find non-use of health care increases households’ risk of illness or death, not only from COVID-19, but also from other health-related problems. As a result, these measures could exacerbate issues such as asthma, access to medication, and broader access to care, [15] find that containment procedures are a barrier to health care utilization as many fear hospitalization [15]., shows that gender is one of the determinants of health care utilization during COVID-19. Hence, it is important to adopt a health equity perspective to address the health inequities that men undergo during pandemics.

In contrast, the supply-side approach to health care is characterized by the availability and characteristics of health services that influence the demand for health care, in other words, the nonexistence of the supply of care, its insufficiency determine the demand for care by households during COVID-19. As classical economics assumes, supply therefore creates its own demand. The choice of care by households depends primarily on the efficiency and quality of health care providers, waiting time, and the cost of services.

The COVID-19 pandemic threatens to disrupt essential health services due to supply and demand barriers. As a result, infant and maternal mortality may increase over the next 12 months. Maintaining essential health services during the COVID-19 pandemic is critical to prevent adverse consequences and to protect the progress made in recent years in reducing mortality. The COVID-19 pandemic causes mortality and morbidity directly attributable to it. It also poses a significant risk to other preventable and treatable diseases if the sufpply of essential health services is interrupted.

Since the beginning of COVID-19 in Togo, policy makers and health care providers have been concerned with the drop in hospital attendance all over the country. Initially, this sharp decline was attributed to the deprogramming of non-emergency care that the majority of households suffer all the time. Then, the confinement, doubled by the fear of contamination in public hospitals could have dissuaded many people not to be presented in case of illness. Needless to say, the care and overload of health care providers have mobilized in large numbers for the sole cause of COVID-19. The consequences of such a situation could be dramatic in terms of public health.

Such a disruption could stem from both supply and demand factors for health care. In Togo, on the supply side, staff providing essential health services are mostly redirected to other health facilities to meet COVID-19 requirements, and many health workers could fall ill or die. Finally, global supply chains for supplies and equipment may be disrupted due to the shift in production to COVID-19-related supplies. All of these lead to decreases in production of raw materials and significant delays in delivery times due to transportation and movement restrictions enacted by policymakers. In Togo, during the pandemic, many immunization programs for children and pregnant women were suspended and this had an impact on the health status of households. In addition, households find it difficult to go to health centers in case of illness for fear of being infected. This mistrust among households accentuates the morbidity rate in the face of difficulties in accessing the various health services. In addition, the rate of access to health facilities in Togo is very high in public health centers, followed by private health services, despite a good number of households that rely on self-medication/traditional care. With this pandemic, most households, lacking financial means and being afraid to go to the public health centers that house people with COVID-19, practice self-medication. This is due to direct and indirect costs related to the demand for care in private health centers. Hence the low demand in both public and private health facilities. The magnitude of COVID-19 limits the management of patients suffering from non-communicable diseases by health care providers in the health services. This raises a serious public health issue, as there is no reason why health problems should have decreased to the extent that they have during this pandemic. The main risk for households is that their health status will deteriorate. However, an increase in late hospitalizations among patients and excess mortality, stemming from postponements of care and consultations with health personnel worsen their health status.

Facing these problems of household health status, they seek inexpensive health care and health centers that do not house patients with COVID-19. Since empirically, certain socioeconomic factors are correlated with household demand for care, they are forced to make a choice among health care providers. With respect to ownership of health care facilities, the available data show a major use of health care by the public and private sectors. Thus, health care utilization diversified in Togo during this pandemic. Most notably, some individuals opted for public sector health facilities. Others chose private health facilities, and a significant proportion of households opted for self-medication. From all the above, there is a need to find answers to the problems posed, hence the following research question: What is the choice of health care providers by households in using health care during COVID-19?

The purpose of this study is to analyze the effect of COVID-19 on the socioeconomic factors that determine the choice of health care providers. Specifically, it will:

- identify socioeconomic factors of health care demand during COVID-19,
- analyze the effect of COVID-19 on health care provider choice during COVID-19.

The results contribute to improve the understanding of this pandemic and trigger actions to reduce the non-COVID-19 mortality rate in Togo. In light of the above, this study examined the factors that determine the choice of health care provider during COVID-19. This article is structured around three parts: Firstly, we will present a methodological approach, followed by the data used. Secondly, we come up with the descriptive analysis of the variables, the results obtained and the discussion of the results. Finally, we offer a conclusion and policy implications.

## Methods

### Conceptual framework for the effect of COVID-19 on health care demand

**Fig. 1:**
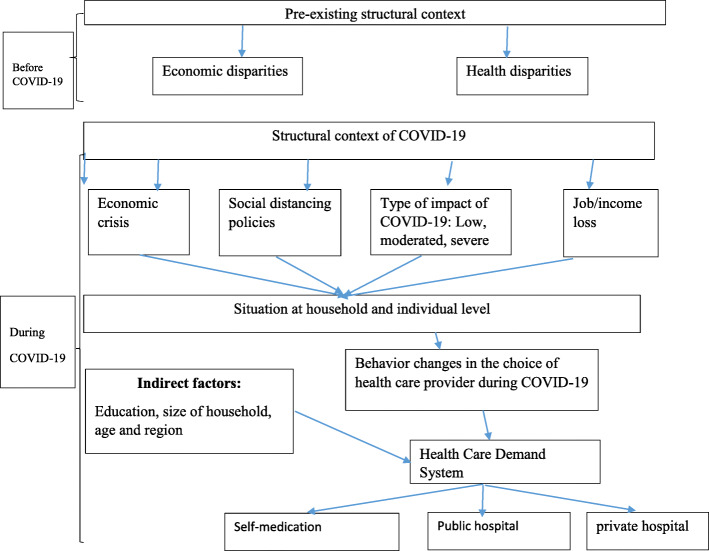
Conceptual framework for the effect of COVID-19 on health care demand

It’s critical to understand the complex pathways by which pandemic-related economic and public health crises contribute to health care demand. We propose a conceptual model of the effect of the COVID-19 pandemic on health care demand that is complex, multilevel, and bidirectional. Conceptual models are useful for establishing complex relationships and facilitating analysis of hypothesized causal associations related to multidimensional health problems. Our conceptual model (Fig. 1) illustrates the multilevel factors (structural, familial, and individual) that influence health care demand during the COVID-19 pandemic, building on previous work [16–18].

In the structural context (Fig. 1), the effect of the COVID-19 pandemic on health care demand is set against the backdrop of pre-existing economic and health disparities. Prior to the COVID-19 pandemic, households as in most low-income countries experience a disproportionate burden as a result of illness [19]. These disparities are due to socioeconomic factors such as employment, household income, education, age and access to health care etc. [20, 21]. Thus, it is in the context of existing health and economic disparities that we can begin to understand how the economic and health crises of COVID-19 intersect at the household level, exacerbating existing disparities.

Analysis of this Fig. 1, shows that in addition to the structural context that existed before the pandemic, COVID-19 introduced its own structural factors. The model proposed here traces the different effects of COVID-19 on socioeconomic factors such as job loss, income, and the types of impact of COVID-19 on household activities that in turn affect household demand for health care. This has a direct effect on household decision making in choosing health care providers. For a loss of employment negatively impacts income and in turn influences the change in household health care demand behavior. Hence the individual may choose public, private or self-medication health care. In addition, indirect factors such as education, household size, age, and region influence household demand for health care during this COVID-19 pandemic. A good level of education allows for an understanding of the effects of COVID-19 and the measures to be taken. In addition, during this period of the COVID-19 pandemic, age is a determining factor in the demand for health care. Individuals of a higher age have a higher risk of becoming infected, hence the need to choose a health care provider or to self-medicate. The analysis of this conceptual card allows us to formalize a specific model on the demand for health care during this pandemic.

### Model specification

During this COVID-19 pandemic, the choice of health care provider by households is a decision following the distrust of health facilities, especially public health centers that house patients living with COVID-19. This choice is made based on quantitative choice models. In this paper, we assume a heterogeneous group of homes *n* consists of n types of homes or groups of patients. Let A, the group of patient, be structured as follows: *i* = 1, 2, 3, , , *n*.

Let us assume that during this period of COVID-19, in case of illness, a home would seek help from a health care provider in a health care center characterized by many providers. The home is supposed to choose medical care from four types of health care providers known as (E), defined by j = 1, 2, 3 with:

✓ *E*_1_: Self-treatment (including traditional medical treatment);

✓ *E*_2_*:* Public hospitals;

✓ *E*_3_: Private hospitals (including mission hospitals).

The probability that a particular alternative will be chosen is equal to the probability that this choice will yield the most utility among all alternatives. Thus, the maximum expected utility of each treatment option for homes is conditional on the characteristics of the health care services associated with each treatment option [E1, E2, E3] and the socioeconomic characteristics of the home or patient who made the choice at one of the health care facilities.

A health care provider is characterized by V, a set of health service system and access variables, with V = (v1, , , , , vd). For a patient, the utility of visiting a health care facility requires making a choice among health care providers during this COVID-19 pandemic. The benefits of each health care provider are determined by the interaction between the characteristics of the health care provider and those gained by the patient.

The presentation of home characteristics are described as follows:

(X) denotes individual home characteristics with *X* = (*x*_1_, *x*_2_, *x*_3_………*x*_*q*_);

(Y) presents the health status characteristics of homes, which at these points of time, during the COVID-19 pandemic, have an actual need to seek or not seek health care services at a health care facility, with *Y* = (*y*_1_, *y*_2_, *y*_3_………*y*_*r*_);

Individuals within the home, require a utility function defined in the context of health care providers, which are conditioned by the socioeconomic factors that determine health care utilization with a health care provider. This choice of health care provider allows him to maximize utility through the quality of health care. Thus, we have:

2 $$ {U}_j\left(\ {X}_i,{Y}_i,{V}_i\right)=\max \left({u}_{i1},{u}_{i2},{u}_{i3},{u}_{i4}\right) $$

Where *u*_*ij*_ = ∝_*j*_*X*_*i*_ + *β*_*j*_*Y*_*i*_ + *φ*_*j*_*V*_*j*_

The parameters ∝_*j*_, *β*_*j*_, et *φ*_*j*_ are vectors of the main variables *X*_*i*_, *Y*_*i*_, *et V*_*i*._

From Eq. 2, the first two parameters are individual characteristics and the last specifies the choice of health care provider. In the framework of the logit model an increase in the variable *X*_*i*_ of an individual defined as i, will change the utility of each home when choosing a health care provider j. On the other hand, an increase in the variable *V*_*j*_ in health care utilization defined as j, will change the utility of home known as i when choosing the health care provider.

These parameters are estimated by applying logit and multinomial model estimation techniques. Thus, Eq. 2 is equivalent to writing:

Thus, Eq. 2 is written as:

3 $$ {U}_{ij}={\propto}_j{X}_i+{\beta}_j,{Y}_i+{\varphi}_j{V}_j+{\varepsilon}_{ij} $$

A home will choose provider defined as j = m if only it offers, among all available choices, the highest level of utility. Thus, if Fi is a random variable whose value is (j = 1, 2, 3, 4, 5) indicates the choice of provider by homes known as i, the probability that person under the index of i will choose alternative (m) is:

4 $$ {P}_r\left({F}_i=m\right)={P}_r\ \left({U}_{im}<{U}_{ij}\right)\kern0.5em \mathrm{with}\ j=1,2,3\ \mathrm{ou}\ j\ne m $$

So *P*_*r*_(*U*_*im*_ + *ε*_*im*_ > *u*_*ij*_ + *ε*_*ij*_) and ${P}_r\left(\overset{\sim }{\varepsilon_{ij}}-{\varepsilon}_{im}<{u}_{im}+{u}_{ij}\right)$

For *j* = 1, 2, 3, with j ≠ m, McFadden [22] showed that if the three error terms ɛij are independent and identically distributed, then with Weibull the distribution becomes:

5 $$ {\displaystyle \begin{array}{c}F\left({\varepsilon}_{ij}\right)=\exp\ \left[\exp \left(-{\varepsilon}_{ij}\right)\right],\\ {}\mathrm{Then}\ {P}_r\ \left({F}_i=m\right)=\frac{\exp \left({U}_{im}\right)}{\sum_{j=1}^3\exp \left({U}_{ij}\right)}\end{array}} $$

The most widely used qualitative choice model is the logit and since the patient’s alternative choices are more than two, a multinomial logit model was adopted for this paper. Each of the N observations on the dependent variable Fi (i = 1,,,,, n) is treated as a single draw from a multinomial distribution with three outcomes, defined as a dummy variable *δ*_*ij*_ = 1 if home known as i made choice j and *δ*_*ij*_ = 0 otherwise, j = 1, , , 3.The likelihood function is then:

6 $$ LogL={\sum}_{i=1}^m{\sum}_{j=i}^3{\delta}_{ij}\mathit{\Pr}\left( Fi=j\right) $$

Based on the application of the multinomial logit model, the probability of household going to the hospital and choosing a health care staff report the self-medication or traditional option, is expressed as follows:

7 $$ \ln \left({P}_r\left({E}_2\right)/{P}_r\left({E}_1\right)\right)=\hat{\theta_2}+\hat{\alpha_2{X}_i}+\hat{\beta_2}{Y}_i+\hat{\varphi_i}{V}_2 $$

8 $$ \ln \left({P}_r\left({E}_3\right)/{P}_r\left({E}_2\right)\right)=\hat{\theta_3}+\hat{\alpha_3{X}_i}+\hat{\beta_3}{Y}_i+\hat{\varphi_i}{V}_5 $$

9 $$ \ln \left({P}_r\left({E}_3\right)/{P}_r\left({E}_1\right)\right)=\hat{\theta_5}+\hat{\alpha_5{X}_i}+\hat{\beta_5}{Y}_i+\hat{\varphi_i}{V}_5 $$

The variables X, Y and V are specified as follows: X is household characteristics (is a vector of individual characteristics such as age, disease severity, education, gender, religion, etc.,), Y is disease perception characteristic (is a random variable, which represents unobserved individual characteristics such as disease severity and complexity that may affect the marginality of productivity providers compared to self-care); V is the characteristic of health services (is a vector of the characteristics of the j facing individual i, these included proximity to health centers, probability of being seen by a doctor, quality of service etc.).

In this study, the multinomial logit model is used because we assumed that the alternative options provide choices, have different attributes and can be considered mutually exclusive. This is consistent with almost all studies that focus on provider choice, they use the multinomial logit method.

### Data source

Empirical studies on the demand for health care are conducted using data collected from households. This survey provides data to optimize the response measures against the spread of COVID-19 put in place by the Togolese government. This data collection on the perspectives of the response against the COVID-19 pandemic in Togo is descriptive and analytical and is conducted on the acceptability, feasibility and practicability of the measures to fight the spread of COVID-19 in Togo. The target population is the Togolese population living in urban areas. The households surveyed are all the people living in the main towns of the health districts. The selection of households is exhaustive and is carried out among the populations of the forty-four (44) districts of the six (6) health regions of Togo. This will ensure comparability between health regions. The analysis of these provide useful information on the demand for health care by household during this COVID-19 pandemic. All of these will help understand the implications of health care utilization during the COVID-19 pandemic.

### Analysis of the descriptive variables

#### Dependent variable

It is the choice of health care providers. The alternative health care providers are: health care providers in public health centers, health care providers in private health centers and self-medication or the fact that households do not make consultations in modern health centers. The choice of health care provider during this period of COVID-19 is done in three steps namely:

- 1 = if no modern health care provider was consulted by the household or self-treatment,
- 2 = if the household was seen by a public health care provider
- 3 = if the household was consulted by a private health care provider.

#### Lists of independent variables used

The independent variables include household socioeconomic factors and are shown in the Table 1.

**Table 1:** Descriptive analyses of variables by health care utilization during COVID-19

Variables	Measurement of variables	Terms and conditions	Percentage (%)
**Sick households**	0. No 1. Yes	No	69.51
		Yes	30.49
**Care provider**	0 Self-Medication 1. Private 2. Public	Self-medication	61.41
		Private	21.20
		Public	17.39
Gender	0. Male 1.Female	Female	56.31
		Male	43.69
Age	0. 18–34 years 1. 35–6 0 years 2. 60 years and over	18–34 years old	41.98
		35–60 years old	44.71
		60 years old and over	13.31
Money transfer	0. No Beneficiary 1.Beneficiary	No Beneficiary	5.80
		Beneficiary	94.20
Family benefit	0. Benefit 1, Occupational risk 2.Health insurance 3.insurance 4.none	No Beneficiary	97.44
		Beneficiary	2.56
Marital status	0. Married 1. Single 2. Divorced 3.Widowed	Single	19.36
		Married	74.60
		Divorced/Widowed	6.04
Education	0. Not in school 1. Primary 2. Secondary 3. Higher	None	19.11
		Primary	23.21
		Secondary	45.39
		Superior	12.29
Loss of employment	0. No 1.Yes	No	81.82
		Yes	18.18
Region	0. Savane 1.Kara 2.Centrale 3.Plateaux 4.Maritime 5. Lomé-commune	Lomé-Commune	7.51
		Maritime	15.53
		Plateaux	25.43
		Central	12.63
		Kara	26.62
		Savanes	12.29

#### Analysis of health care utilization characteristics of COVID-19

The survey results reveal that among 30.49% of households that were consulted ill during COVID-19, 61.41% of households practiced self-medication, 21.20% used private health centers against a low proportion of 17.39% who used public health care facilities. This low proportion of the use of public health centers can be explained by the fact that patients with COVID-19 are housed in these health centers. Hence, the high risk of homes to use them.

Table 1 presents the descriptive statistics of the variables related to the risks of health care seeking by households during COVID-19. An analysis of the table shows that about 56.3% of the households surveyed are women took the risk of seeking health care compared to 43.6% by men. Among these households, 19.3% have no level of education, 23.2% have a primary level of education and 12.2% of these households have reached a higher level of education have used health care. On the other hand, the majority of the surveyed households with secondary level (45.3%) were able to use health care during COVID-19.

Approximately 97.44% of respondents who did not receive family benefits claimed to use health facilities during the pandemic, compared to 2.56% who received family benefits used health care in Togo. This is consistent with the widespread perception of the negative impacts of the COVID-19 health crisis on household demand for health care. In addition, the statistics highlight the behavior of households with respect to health care demand following the loss of their jobs during this pandemic. Thus, 18.18% of homes that lost a job were able to use health care centers whose incomes were impacted by Covid-19 on health care demand. In addition, the cash transfer program dedicated to the most vulnerable homes was only accessible to a small proportion of beneficiaries. Among this proportion of homes, 94.2% of the surveyed homes receiving these cash transfers used health care when they were sick.

#### Choice of health care providers during COVID-19 by level of education

The analyses in this table show that most households reduce the risk of becoming contaminated in public health facilities by opting for self-medication as the level of education increases. So, this frequency table below shows that more than half of the 26 (72.22%) households with no education level prefer self-medication during the COVID-19 pandemic, while this proportion is 5 (13.89%) for both public and private health care providers. This low proportion is explained by the fact that public health facilities are sheltered by patients with COVID-19. Nevertheless, 27 (54%) of primary level patients opt for self-medication compared to 13 (26%) in private health centers and 10 (20%) in public health centers. In addition, with a higher level of education the proportion is high 19 (65.52%) for self-medication, 4 (13.79%) prefer private health centers and 6 (20.69%) use public health providers. This high proportion of self-medication is related to the loss of employment of households during the COVID-19 pandemic and the risk that these individuals may be contaminated.

#### Choice of health care providers during COVID-19 according to certain social protection characteristics

Households were allocated according to the choice of health care providers during COVID-19 via certain variables such as loss of income, health insurance and family benefit. The results survey reveals that households with employment accounting for 81 (65.85%) have a higher proportion of self-medication per risk of being contaminated by COVID-19. On the other hand, in the same situation, 21 (17.07%) of employed households have a preference for using public and private health centers in the event of illness. In addition, the analysis shows that households with social protection such as health insurance and family benefits have a high proportion of self-medication. This proportion is respectively 17 (54.84%) for health insurance and 2 (50.00%) for health care providers. On the other hand, on behalf of the health insurance, 3 (54.84%) of households with insurance choose private health centers compared with 11 (35.48%) for public health centers. With regard to the choice of health care providers, households’ preferences seem to shift from self-medication in public to private health care facilities in terms of the level of employment, health insurance and social benefits (Tables 2 and 3).

**Table 2:** Choice of health care providers during COVID-19 by education

Education	Choice of health care provider during COVID-19		
	Self-medication Number (%)	Private Number (%)	Public Number (%)
None	26 (72.22)	5 (13.89)	5 (13.89)
Primary	27 (54.00)	13 (26.00)	10 (20.00)
Secondary	44 (60.27)	17 (23.29)	12 (16.44)
Superior	19 (65.52)	4 (13.79)	6 (20.69)

**Table 3:** Choice of health care providers during COVID-19 according to social protection characteristics

	Choice of health care provider during COVID-19		
	Self-medication Number (%)	Private Number (%)	Public Number (%)
Employment during COVID-19			
No loss	81 (65.85)	21 (17.07)	21 (17.07)
Loss of employment	19 (61.29)	6 (19.35)	6 (19.35)
**Health insurance**			
No insurance	99 (63.06)	36 (22.93)	22 (14.01)
Benefit from insurance	17 (54.84)	3 (54.84)	11 (35.48)
**Family benefit**			
No services	114 (61.96)	38 (20.65)	32 (17.39)
Benefit from a service	2 (50.00)	1 (25.00)	1 (25.00)

## Results

The Multinomial logit model is used to estimate the choice of care providers when the household wants to seek care or not during this COVID-19 pandemic. The variables used are derived from work [11–13] which classified socio-economic factors that influence the demand for health care services into three categories: predisposing factors, enabling factors, and severity of illness. The results are presented in Table 4.

**Table 4:** Analysis of the choice of health care provider during COVID-19

Variables	Self-medication				Public hospitals			
	RRR	*P*-value	95%Conf	Interval	RRR	P-value	95%Con	Interval
**Gender**								
Male	0.61	0.38	0.2	1.85	0.79	0.69	0.28	2.55
**Age**								
35–60 years old	0.94	0.92	0.28	3.17	0.59	0.40	0.17	2.04
More than 60 years old	23.97*	0.06	0.93	615.38	12.59	0.13	0.47	334.492
**Size of household**								
4–6	0.81	0.80	0.16	4.05	0.48	0.40	0.09	2.68
6 and more	0.17**	0.03	0.03	0.85	0.25	0.12	0.04	1.42
**Marital status**					1			
Married	0.42	0.27	0.09	1.99	1.17	0.86	0.19	6.89
Widow (widower)	0.73	0.84	0.07	14.85	1.09	0.96	0.04	30.11
**Education**								
Primary	0.99	0.99	0.19	5.00	0.98	0.98	0.17	5.63
Secondary	0.47	0.29	0.11	1.95	0.42	0.28	0.09	2.02
Superior	0.18	0.14	0.02	1.77	0.19*	0.018	0.02	2.11
**Region**								
Maritime	2.96	0.33	0.34	26.01	21.31	0.03	1.3	349.52
Plateaux	5.95**	0.10	0.71	49.92	4.43	0.32	0.24	83.03
Central	15.9***	0.02	1.49	169.83	6.92**	0.24	0.28	173.43
Kara	195.9**	0.003	12.43	3089.85	559.48***	0.25	20.64	1516.3
Dapaong	19.93	0.04	1.12	356.18	131.44**	0.01	4.68	3691.6
Health insurance	0.56	0.35	0.17	1.89	1.05	0.94	0.28	3.92
Loss of employment	6.94***	0.01	1.59	30.24	4.89***	0.05	1.02	23.38
Assets before COVID-19	4.92***	0.01	1.52	15.89	3.14***	0.07	0.91	10.83
**Impact COVID-19**								
Low	100,556	0.99	0.001	1.26	248,821	0.00	0.01	1.53
Moderated	0.10*	0.06	0.01	1.12	0.19	0.98	0.017	2.23
Severe	0.12*	0.07	0.012	1.16	0.42	0.19	0.04	4.32
Constant	0.53	0.65	0.03	8.43	0.12	0.74	0.04	3.51
Mean dependent var	1.90	SD dependent var.	0.88					
Pseudo r-squared	0.29	Number of obs	171.00					
Chi-square	105.21	Prob > chi2	0.000					
Akaike crit, (AIC)	354.14	Bayesian crit, (BIC)	504.94					

*** p < .01, ** p < .05, * p < .1

Relative risk ratios (RRR) determined from multinomial logit regression indicate the risk that individuals face when choosing a survival strategy relative to the choice of the reference modality. The aim is therefore to compare relative risk ratios of, for example, using one type of care rather than another. Thus, the household is considered to remain in its strategy if RRR > 1 and to choose the reference modality if RRR < 1. Thus, the statistical significance of the coefficients of certain explanatory variables enables us to identify the variables which explain the choice of households to use a given care structure compared to the reference health structure (public hospital). Generally speaking, the results indicate that households prefer private hospitals to public hospitals, since for many explanatory variables the relative risk ratio is less than 1.

Age is positively related to the demand for public health services and private sector hospitals. On the other hand, the coefficient of the relative risk ratio is significant and greater than 1. Age is also found to be a factor in explaining the choice of health care use by households during the pandemic. Indeed, compared to young households aged between 18 and 34, individuals aged 60 and over take the risk of remaining in their choice of self-medication in favor of private hospitals. This implies that as their age increases, they tend to self-medicate because they are limited by measures of the response to COVID-19 such as social distancing, inter-city travel, and mask purchase. This reduces health care costs if they agree to use the health center. However, older people prefer modern health facilities because of their state of health. This continued self-medication by older people is explained by the fact that specialized services are housed in public hospitals. However, during this health crisis, public hospitals are overwhelmed by patients with COVID-19.

On the other hand, when referring to household size, the weighting factor of the risk ratio is significant and less than 1, implying that the size of the household determines the choice made per household during COVID-19. It appears that the latter influences the risk taking of modern (private) care more than public care. The preference for those households with more than 6 children is explained by the fact that these households are no longer able to control in case of illness, hence the use of modern health care.

In terms of the choice of health care providers, the educational level of households is also positively related to the demand for care. The weighting factor of the education level variable is significant and the relative risk ratio is less than 1. This implies that during the COVID-19 pandemic, individuals with a high level of education prefer to use private health center rather than self-medication or a public health center, all of which removes them from the risk of contamination if they were to use these health center. Indeed, it appears that the higher the level of education of individuals, the more they have a preference for private hospitals instead of self-medication and public hospitals. Also, households with a higher level of education tend to maintain their habit of using private health care during the pandemic period. In all cases, the relationships were significant at 1%. This implies that educated households tend to use private hospitals more than other health center. This observed result is due to the perceived quality of care as well as the availability of specialized services provided by private hospitals. Finally, during this COVID-19, it is observed that the choice of private health center was influenced by the level of education of the households, but it influences the choice of a private facility.

Regarding the perception of the effects of the response measures at the household level, the results reveal that households that experienced a moderate and severe effect from Covid-19 preferred an alternative to using a private health center rather than self-medicating or using public health facilities. This is because the coefficient of moderate and severe effects is significant and less than 1. All in all, the impact of Covid-19 affected the choice of health care providers when households fell ill during Covid-19. Therefore, these households are at high risk of using public hospitals, which in most cases, house public health center.

Furthermore, the weighting factor of the relative risk ratio of household activities before COVID-19 is significant and greater than 1 in the choice of health care provision such as self-medication and household use of public health care. Those households lost their jobs to remain in the choice of food consumption rather than eating as usual.

The results also indicate that individuals residing in the Plateaux, Centrale and Kara region show preference to self-medication. On the other hand, households living in the Central, Kara and Savane region remain in their choice of using public health center compared to the Maritime or Lomé-commune region, where the relative risk ratio weighting factors are greater than 1, as these households did not want to change their choice of health care provision during the pandemic. These results can be explained by the fact that these regions are not infected by COVID-19 so that policy makers can implement response measures in these regions such as distancing, intra-urban movement.

## Discussion

This study explored the effects of COVID-19 and the experiences of health service utilization during the COVID-19 pandemic in Togo. It shows that the COVID-19 pandemic acts as an external factor to highlight weaknesses in Togo’s recently reformed health care system, particularly how it affects health service utilization. Health service utilization was found to be affected by population characteristics inherent in predisposing factors, enabling factors and perceptions of health services and the current pandemic (attitudes, values, knowledge and understanding). Thus, COVID-19 has affected the existing dynamics of health service utilization, including its stakeholders, such as changes in health worker behavior, politicization of health services and related adversities.

The results of this study show that 61.70% of households used self-medication, 20.74% in private health center and 17.55% in public health center during the pandemic. These results show the low demand for modern health services among the households surveyed, especially the public health facilities that house patients with COVID-19 or households fearing the risk of contamination, hence the low use of health care. This implies, however, that a significant number of households do not benefit from health care services, and they prefer self-medication for fear of being infected with COVID-19. The low demand for care observed by households is due to community health or other prepayment schemes.

Multinomial logit regression analysis revealed that households with a higher level of education increase the likelihood of choosing a modern health care facility than households with no education. This is explained by the fact that educated people have better information about modern health care facilities. In order to respect the barrier measures and the social distancing required during this period, the behavior and attitudes adopted by households towards COVID-19 had economic impacts on the demand for health care. As a result, these households experienced economic shocks on their living conditions. This health crisis and its corollaries affect the loss of employment of households, making it difficult for them to seek care in case of illness. Household job losses that have an impact on the use of health care are due to curfews and the cessation of business and administrative activities. Currently, prohibitions and limitations imposed to combat COVID-19 affect the choice of health care providers. Specifically, the results show that 15.4% of households face problems in accessing health facilities and these households are obliged to purchase alcohol gels and face masks as protective measures. Besides, the pandemic has already had a catastrophic impact on the most vulnerable households. In addition, the pandemic has already had a catastrophic impact on the most vulnerable households. These households have a deteriorating health status in the event of illness are exposed to higher health risks. As a result, they face a greater limitation in access to basic health care.

With regard to the substitution of COVID-19 control products, there is an increase in user fees in private health facilities due to mistrust in public health center. All this reduces the likelihood of using health care at the modern health care provider compared to self-treatment. This could be explained by the fact that the risk of health care demand is affected by the impact of COVID-19 on household income-generating activities, job loss and household education levels. For example, the United Nations (UN, 2020) estimates that the reduction/loss of household income due to COVID-19and the reduction in essential health expenditures could wipe out the progress in mortality reduction over the past three years. In Togo, the most cited effect of the pandemic is the reduction or closure of commercial activities due to restrictions by policy makers. Similar effects of the pandemic on sources of income were reported by households. For example, the total loss of jobs as the economic consequences of the pandemic was reported by households, with 14.16% losing their jobs during the COVID-19 pandemic. The most cited effect of the pandemic was the reduction or closure of business activities due to restrictions by policy makers. These disruptions to income-generating activities induced by COVID-19 have been observed in several reports and studies [23]. All of these have pronounced effects on the demand for health care.

Most households in this study expressed anxiety and fear about COVID-19, not only because of the perception that COVID-19 is a highly fatal disease, but also because of the bias of uncertainty about the pandemic situation in Togo. The level of fear and anxiety varied according to the area of residence, population density. The stigma attached to COVID-19 among community members triggered a well-established mechanism of “hiding” the disease to avoid discrimination, which can nevertheless be detrimental as it can impede early health-seeking behavior. A previous study from 28 countries highlighted that social stigma attached to COVID-19 has led to perceived risks and fears of losing loved ones, facing intolerance, and other discriminatory acts [24]. Moreover, the continued rapid increase in the number of COVID-19 cases accompanied by an “infodemic” might have fueled anxiety and fear among the local population United Nations [25]. In the face of the resounding health care utilization during the pandemic, the Togolese population has also seen and heard how COVID-19 patients are stigmatized in hospitals and can only be visited by their close family members. These multi-faceted acts of discrimination against COVID-19 have added to the fear and stigma of the disease [26]. For example, it has been found that fear and anxiety among health care providers is associated with the scarcity of personal protective equipment and fear of transmission [22]. To some extent, fear of disease in the community may play a positive role in increasing compliance with government-imposed public health measures.

## Conclusions

The use of basic health services during COVID-19 is one of the key factors promoting better health for populations. The literature indicates that analysis of the determinants of health care demand is extremely important for the formulation of policies and strategies in the health sector but also for ensuring effective use of health care services. This study analyses the effect of COVID-19 on the health care demand among households in Togo. The results show a deterioration in household demand for health care during the COVID-19. Socio-economic characteristics such as loss of employment, level of education, place of residence, health insurance and social protection are important factors as regards household demand for health care during this COVID-19 in modern health care settings.

Based on the findings, this paper suggests the following strategies to help stabilize the proportion of household health care accessibility during the COVID-19. Firstly, policy makers need to implement structural changes in social protection mechanisms to facilitate access to care for households regardless of the choice of health care provider. Secondly, there is a need to broaden the coverage of health insurance and family benefits, especially for low-income people and households working in the informal sectors. A better understanding of the determinants of health care demand makes it possible to introduce and implement appropriate incentive systems. All these factors help encourage better use of health care services during pandemics.

## Data Availability

The datasets used and/or analyzed in this study are available from the Bioethics Committee for Health Research (CBRS) from the Togo Ministry of Health (CBRS No004/2020/CBRS) upon reasonable request. Data to support the findings in this article are available from CBRS and may be obtained with written permission. These sites allow you to contact the CBRS to make a request for available databases.

## Abbreviations

UNOPS: United Nations Office for Project Services
COVID-19: Coronavirus disease 2019
WHO: World Health Organization

## Declarations

### Please provide a statement to confirm that all methods were carried out in accordance with relevant guidelines and regulations

I confirm with this note that all the information and methods given have been applied in accordance with the directives and regulations or All protocols are carried out in accordance with relevant guidelines and regulations.’
